# The Role of hnRPUL1 Involved in DNA Damage Response Is Related to PARP1

**DOI:** 10.1371/journal.pone.0060208

**Published:** 2013-04-05

**Authors:** Zehui Hong, Jie Jiang, Jiaolong Ma, Shikui Dai, Ting Xu, Hui Li, Akira Yasui

**Affiliations:** 1 Department of Genetics and Developmental Biology, Medical School of Southeast University, The Key Laboratory of Developmental Genes and Human Disease in Ministry of Education, Nanjing, China; 2 Department of Internal Medicine, Nanjing Municipal Government Hospital, Nanjing, China; 3 Department of Urology, Jiangsu Cancer Hospital, Nanjing, China; 4 Division of Dynamic proteome, Institute of Development, Aging and Cancer, Tohoku University, Sendai, Japan; University of Texas Health Science Center at San Antonio, United States of America

## Abstract

Heterogeneous nuclear ribonucleoprotein U-like 1 (hnRPUL1) -also known as adenovirus early region 1B-associated proteins 5 (E1B-AP5) - plays a role in RNA metabolism. Recently, hnRPUL1 has also been shown to be involved in DNA damage response, but the function of hnRPUL1 in response to DNA damage remains unclear. Here, we have demonstrated that hnRPUL1 is associated with PARP1 and recruited to DNA double-strand breaks (DSBs) sites in a PARP1-mediated poly (ADP-ribosyl) ation dependent manner. In turn, hnRPUL1 knockdown enhances the recruitment of PARP1 to DSBs sites. Specifically, we showed that hnRPUL1 is also implicated in the transcriptional regulation of PARP1 gene. Thus, we propose hnRPUL1 as a new component related to PARP1 in DNA damage response and repair.

## Introduction

Heterogeneous nuclear ribonucleoproteins (hnRNPs) are a large family of proteins, which were first described as a set of proteins binding to mRNA in the cell nucleus. The hnRNPs are multifunctional proteins involved in many cellular processes such as chromatin remodeling, transcription and telomere elongation [1], [2]. Heterogeneous nuclear ribonucleoprotein U-like 1 (hnRPUL1) is also named as adenovirus early region 1B-associated proteins 5 (E1B-AP5), for it was firstly isolated from a human expression library as interacting protein with adenovirus early protein E1B-55 kDa (Ad5EE1B55K) [3]. On the basis of sequence similarity to hnRNP U and hnRNP G, hnRPUL1 is considered to be a new member of hnRNP family. Like other hnRNPs, hnRPUL1 plays a role in RNA metabolism, which binds to mRNA and influences RNA transport and processing [3]. HnRPUL1 is also associated with TAP which is involved in RNA export [4].

In addition to function in RNA metabolism, hnRPUL1 is also involved in other cellular processes. In recent study, hnRPUL1 was identified to be associated with early-onset myocardial infarction (MI) [5]. In another study, hnRPUL1 was demonstrated to interact directly with p53 and inhibit p53 transcriptional activity in response to UV radiation. Furthermore, hnRPUL1 is required for the ATR-dependent signaling in response to viral infection; these studies suggested that hnRPUL1 plays a role in DNA damage response [6].

DNA damage response and repair contribute to maintain genome stability. Cellular DNA is vulnerable to all types of DNA-damaging agents, including both endogenous and environmental origins. A human cell could generate over 10^4^ DNA lesions per day [7]. For maintaining genome stability, cells have evolved a series of mechanisms to repair all types of DNA lesions, such as base damage, single strand-breaks (SSBs) and double strand-breaks (DSBs) [7], [8]. DNA damage response and repair is a complex process, which involves a series of signal transduction pathways including repair, cell cycle regulation, chromatin remodeling and apoptosis. All components of these pathways are composed of sensors, mediators and effectors [9]. PARP1 is one of the most important proteins required to maintain genome stability. Like ATM, ATR, DNA-PK and p53, PARP1 binds to and is activated by DNA strand breaks. Subsequently, PARP1 poly (ADP-ribosyl) ates itself and other proteins to regulate DNA damage response and repair [10], [11].

Although previous studies have shown that hnRPUL1 plays a role in DNA damage response and repair, the function of hnRPUL1 in response to DNA damage remained unclear. In this study, we provide new insights into the function of hnRPUL1 in response to DNA damage and for repair. We have established various types of real-time analysis methods that can induce SSBs and DSBs at restricted regions in the nucleus and can analyze the response of proteins to damage by immuno-staining or with transfected EGFP-tagged proteins under the microscope in real time [12], [13]. By making use of these methods, we showed that hnRPUL1 is associated with PARP1 and recruited to damage sites which are regulated by poly (ADP-ribosyl) ation. Downregulation of hnRPUL1 reduces PARP1 expression by transcriptional regulation and causes cell sensitivity to DSBs damage. Our data indicated that hnRPUL1 plays an important role in response to DNA damage, thereby maintaining genome stability.

## Materials and Methods

### Construction of Plasmids for Expression of Various Genes

Plasmids expressing human genes encoding PARP1 were constructed by cloning cDNA amplified from HeLa cells. Two transcript variants (isoform a and isoform d) encoding different isoforms have been found for hnRPUL1 gene. Isoform d (also named variant 4) differs in the 5′ UTR and coding region compared to isoform a (also named variant 1), resulting in an isoform (d) that maintains the reading frame but is shorter at the N-terminus compared to isoform a. HnRPUL1 (isoform d) was amplified from a human cDNA clone (no. : FLJ12944) and used in experiment of this study. We modified the multiple cloning sites of vectors EGFP-C1, EGFP-N1 to introduce various cDNAs attached with an in-frame *Xho* I or *Sal* I site at the start and *Not* I site at the stop codons. Deletion fragments of hnRPUL1 were generated by PCR amplification, and then cloned into vectors. All constructs were verified by sequencing.

### Cell Lines, Culture and Transfection

The following cell lines were used in this study: HeLa, MEF, MEF (PARP1−/−), Flp-In-293 (Invitrogen), XPA-UVDE (For UV-induced SSB production, XP12ROSV cells stably transfected with the UVDE gene from *Neurospora crassa*). U2OS/TRE/I-SceI-19 (for I-SceI-induced DSB production). All cell lines were propagated in Dulbecco’s modified-MEM (Nissui), supplement with 1 mM L-glutamine and 10% fetal bovine serum at 37°C and 5% CO_2_. For UVA-laser irradiation, cells were plated in glass-bottomed dishes (Matsunami Glass) and transfected with expression vectors using fuGeneHD (Roche), according to the manufacturer’s protocol.

### Microscopy and UVA-laser Irradiation

Fluorescence images were obtained and processed using a confocal scanning laser microscopy system (FV-500, Olympus). UVA-laser irradiation was used to induce DSBs in cultured cells as described previously. For laser irradiation, cells were treated with 10 mM 5-bromo-2-deoxyuridine (BrdU) for 24 hr prior to irradiation. The irradiation dose was fixed at 800 mW (500 scans with 1.6 mW/scan) for BrdU-treated cells. For evaluation of accumulation kinetics, recruitment of EGFP-PARP1 was measured as the fold increase of fluorescence intensity at an irradiated site, at least four independent experiments are given. To examine the effect of inhibitors of PARP, cells were incubated for 1 h in medium supplemented with 1,5-dihydroxyisoquinoline (DIQ; 500 uM; Sigma) or 3-aminobenzamide (3-AB; 4 mM; Sigma) before irradiation.

### Survival Assay

About 400 HeLa cells were plated on 6 cm dishes. For treatment with *cis*-diamminedichloroplatinum(II) (CDDP, Nihonkayaku) or methylmethanesulfonate. (MMS, Sigma), cells were incubated in DMEM containing chemicals for 24 hr, then washed twices, and fresh DMEM was added. For irradiation with X-rays, 8 hr after plating cells were irradiated with indicated dose. Eight days later, cells were stained with 0.3% crystal violet, and colonies were counted. Each experiment was performed three times and standard errors were calculated and are indicated in the graphs.

### Immunofluorescence

Cells were fixed in cold methanol/acetone (1∶1) for 10 min at −20°C and probed with rabbit anti-hnRPUL1 (1∶50, A300-863A Bethyl), mouse anti-γH2AX (1∶400; jbw103, Upstate), Mouse anti-PARP1 (1∶100 sc-8007, Santa Cruz Biotechnology). The secondary antibody used was Alexa fluor 488 anti-rabbit IgG and 594 anti-mouse IgG (1∶400; molecular Probes). Nuclear DNA was stained with 4′,6′-diamidino-2-phenylindole (DAPI; 0.5µg/ml, Wako). Fluorescence microscopy was performed using the same microscopy as used in laser micro-irradiation.

### Immunoblotting

Cells were sonicated in SDS buffer and boiled, and then cleared by centrifugation. Proteins were separated by SDS/PAGE, electroblotted, and detected with the following antibodies: rabbit anti-hnRPUL1 (1∶1000; A300-863A, Bethyl), mouse anti-PARP1 (1∶3000; sc-8007, Santa Cruz Biotechnology), mouse anti-FLAG (1∶3000; F-3165, Sigma), goat anti-actin (1∶2000; I-19, Santa Cruz Biotechnology). The secondary antibodies used were from Santa Cruz Biotechnology: anti-rabbit IgG-HRP (1∶3000, sc-2004), anti-mouse IgG-HRP (1∶3000, sc-2005) and anti-goat IgG-HRP (1∶3000, sc-2056).

### Stable Cell Lines

Stable isogenic cell lines expressing hnRPUL1 tagged with FLAG-HA were established using Flp-In system (Invitrogen) as described previously, according to the manufacturer’s protocol. Briefly, we modified pcDNA5/FRT vector (Invitrogen) to introduce various cDNAs attached with an in-frame *Xho* I site at the start and *Not* I site at the stop codons and put two different epitope tags tandemly (FLAG-HA) on the 5′-terminus of cloning site. Flp-In-293 cells were cotransfected with a 1∶9 ratio of pcDNA/FRT:pOG44 and selected with hygromycin. Hygromycin-resistant cell clones were picked up and expanded. Target protein expression was verified by immunoblotting. Control cell line was generated by transfecting Flp-In-293 cells with pcDNA5/FRT blank vector containing Flag-HA tags.

### Local UV Irradiation

Local UV irradiation was performed as described previously (Okano et al., 2003). UV irradiation was delivered using a germicidal lamp (GL-10; Toshiba; predominantly 254 nm) at a dose rate of 1.25 J/m^2^/second. Before UV irradiation, cells were washed once with Hank’s buffer and gently covered with a polycarbonate isopore membrane filter containing 3 µm diameter pores (Millipore). Cells were irradiated locally with UV through the pores.

### Generation of a Stable hnRPUL1 Knockdown Cell Line and Colony Formation Assay

Short hairpin siRNA constructs were designed around 21 nucleotide sequences against hnRPUL1 (5′-GCAGGCCTATCGTCCAGAAAT-3′). Oligonucleotides were synthesized and cloned into the psiRNA-h7SKzeo G1 vector (Invivogen). The hnRPUL1 siRNA vector and control vector (psiRNA-h7SKz-Luc, Invivogen) were transfected into HeLa cells by fuGeneHD (Roche). Stable transfectants were selected in the presence of 500 µg/ml zeocin. Knockdown of hnRPUL1 was detected by immunoblotting. For the colony formation assay, cells were plated in duplicate at 400 cells/6-cm dishes. Eight hours after plating, cells were irradiated with X-Ray. Eight days later, colonies were fixed and stained with 0.3% crystal violet in methanol for counting. Three independent experiments were carried out and the standard errors were indicated with an error bar.

### Quantitative Real-time PCR

RNA was extracted from 30 mg of tumor or normal tissue, and from cultured cells using the TRIzol Reagent(SunshineBio, SN114)according to the manufacturer’s instructions. RNA was retrotranscribed using PrimeScript® RT reagent Kit with gDNA Eraser (Takara DRR047A). Real-time PCR assays were performed using SYBR®Premix Ex Taq™(Takara, DRR420A) and an ABI 7300 Real time PCR system(Applied Biosystems). The primer sequences were as follows: 5′-CAAACAAGAAAACGAGTCAGGC-3′ (forward) and 5′-TCTTTCGACTCTGGAATTGCTG-3′ (reverse) for hnRPUL1, 5′-CTACTCGGTCCAAGATCGCC- 3′ and 5′-TTGAAAAAGCCCTAAAGGCTCA-3′(reverse) for PARP1, 5′-AAAGACCTGTACGCCAACAC-3′ (forward) and 5′-GTCATACTCCTGCTTGCTGAT-3′ (reverse) for β-Actin. RNA expression values were normalized versus actin. Thermal cycling was initiated with a denaturation step of 95 for 30 s and consisted of 40 cycles (denaturation at 95°C for 5 s, annealing and elongation at 60°C for 31 s). All experiments were performed in triplicate. A non-template control was included in each experiment. Melting curve analysis and agarose electrophoresis were carried out to validate the specificity of the amplification products.

## Results

### HnRPUL1 is Recruited to DSBs Sites

**Figure 1:**
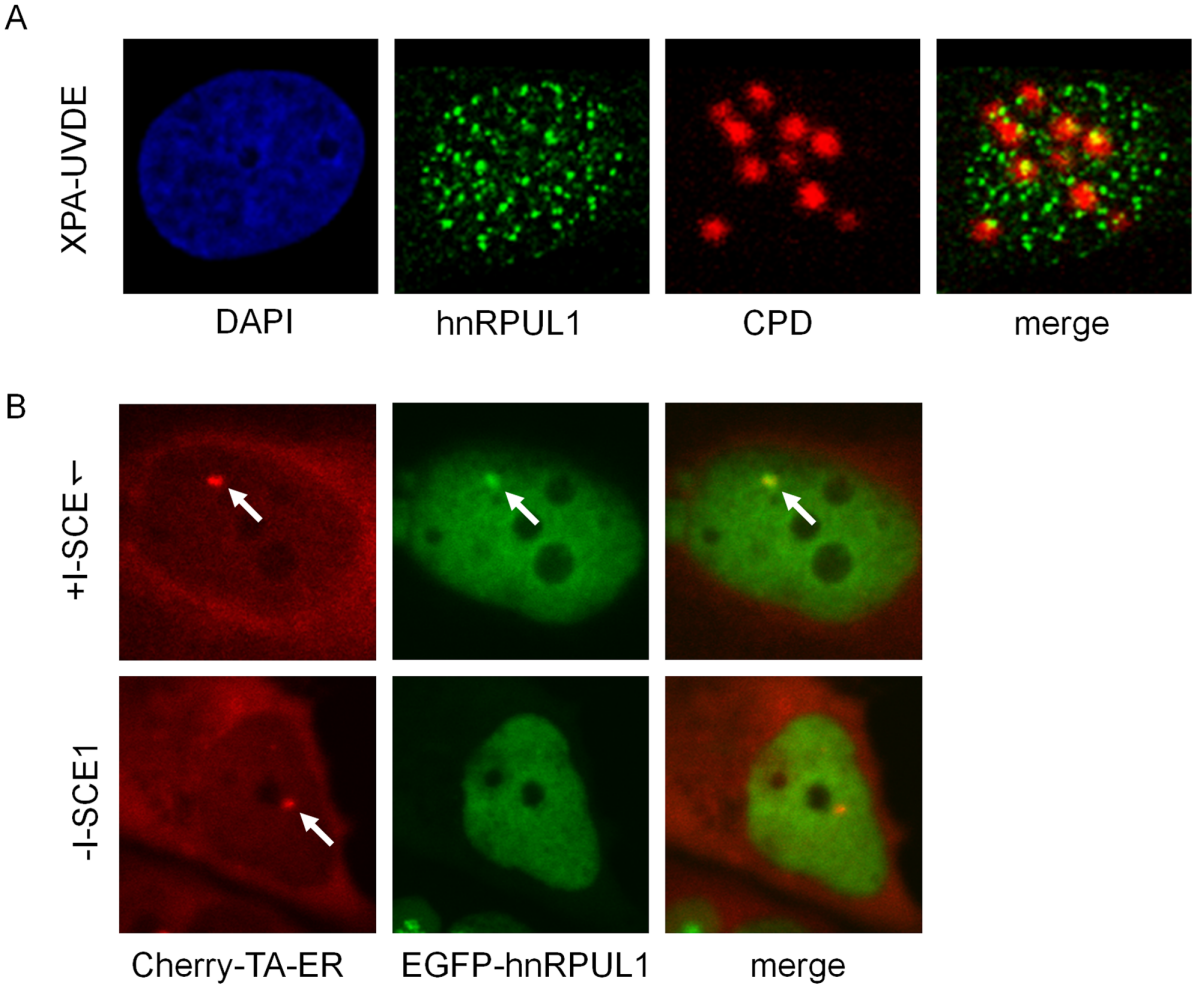
Recruitment of hnRPUL1 to DSBs sites. (A) No recruitment of hnRPUL1 to UVDE-induced SSBs sites indicated by CPD in XPA-UVDE cells after local UV irradiation (60 J/m^2^). (B) Recruitment of EGFP-hnRPUL1 to DSBs sites induced by I-SceI indicated by focus of EGFP-hnRPUL1 colocalized with that of Cherry-tTA-ER after transfection of the plasmid pCMV-NLS-I-SceI (+I-SceI). Arrows indicate the sites of DSBs produced by I-SceI.

Although hnRPUL1 was shown to be involved in DNA damage response, the function of hnRPUL1 is still not clear. Recently, hnRPUL1 was shown to be recruited to DNA damage sites induced by laser [14]. In line with the study, we also found that EGFP-hnRPUL1 (variant 4) is transiently recruited to DNA damage sites in our laser system (Figure S1A). Endogenous hnRPUL1 was identified at the irradiated sites and co-localized with γH2AX in HeLa cells (Figure S1B). Under the condition of laser micro-irradiation used, laser could induce base damage, SSBs and DSBs. To demonstrate to which DNA damage site hnRPUL1 is recruited, we analyzed whether hnRPUL1 is recruited to the multiple sites of DSBs produced by the restriction enzyme I-*Sce*I within human cells [15] and the sites of SSBs induced by the UV damage endonuclease (UVDE) and local irradiation with UV light in XPA-UVDE cells [16]. As shown in figure 1A, while we observed UV-induced foci formation of GFP-hnRPUL1 over the nucleus, the foci do not coincide with the UV-irradiated site, where SSBs were produced by expressed UVDE. In contrast, a clear focus of expressed EGFP-hnRPUL1 was identified at the same position as Cherry-tTA-ER in cells after transfection with pCMVNLS-I-*Sce*I plasmid, where multiple I-*Sce*I sites are present (Figure 1B). These data suggest that hnRPUL1 is recruited to DSBs sites but not to SSBs.

### Recruitment of hnRPUL1 is Mediated by its C-terminus

**Figure 2:**
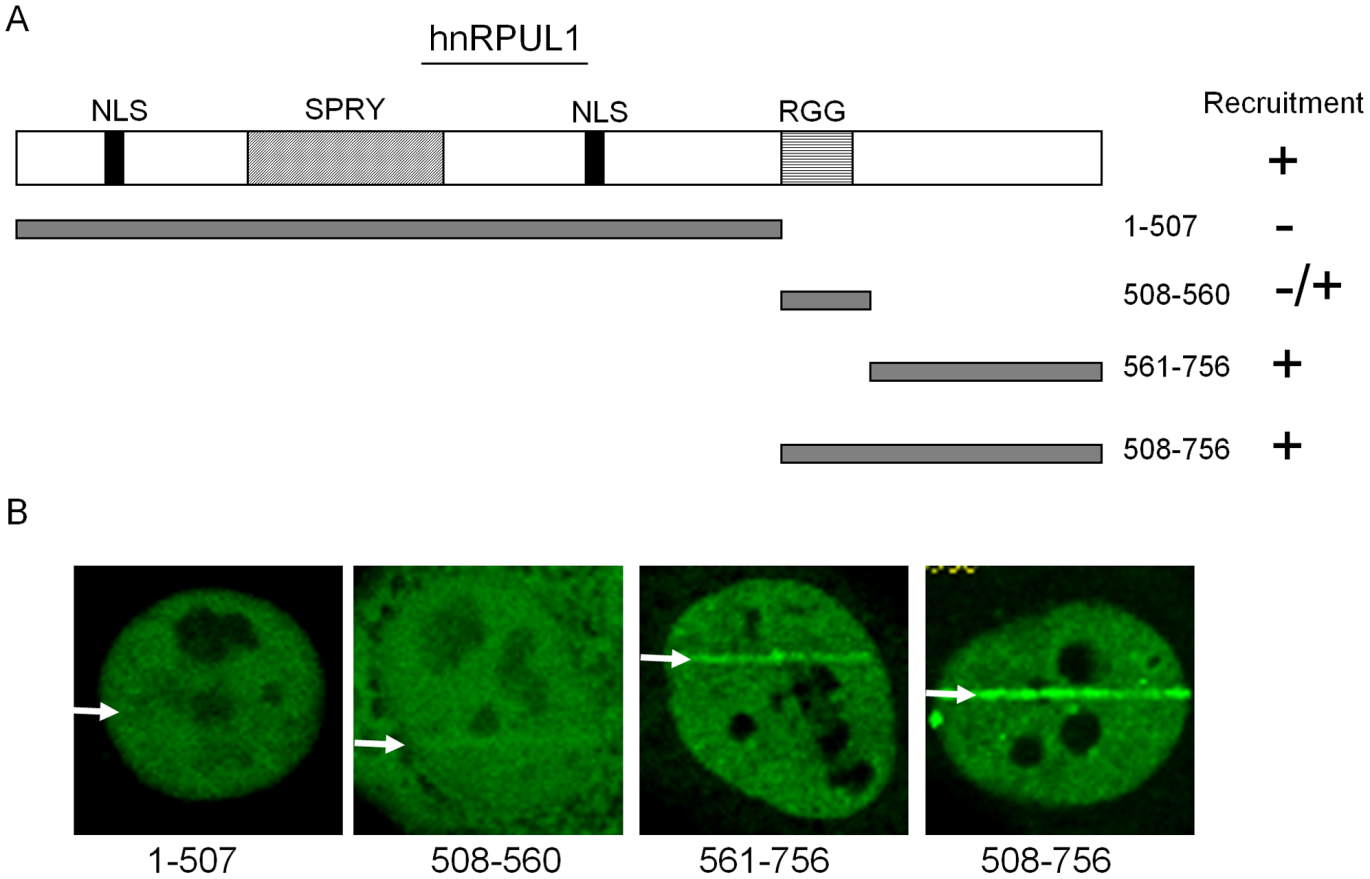
HnRPUL1 is recruited to laser-irradiated sites via the C-terminus (aa 561–756). (A) Schematic presentation of hnRPUL1 domains (top) and deletion mutants (left) and the results of recruitment experiments (right). (B) Live cell imaging of irradiated HeLa cells expressing EGFP-tagged deletion mutants of hnRPUL1. Arrows indicate the sites of irradiation.

The full-length of hnRPUL1 (hnRPUL1 variant 1) contains an RGG motif, a SAP motif and a SPRY motif. The RGG motifs are some Arg-Gly-Gly tripepetide repeats which are involved in RNA binding, protein-protein interactions, transcriptional activation and nuclear localization. It had been shown that hnRPUL1 is also methylated on arginine residues in the RGG motif [17]. The SAP motif has DNA binding activity and is found in diverse nuclear proteins involved in transcription, DNA repair, RNA processing or apoptotic chromatin degradation (in chromosomal organization). The SPRY motif is present in a large number of proteins with diverse individual functions in different biological processes and is likely to function through protein-protein interaction. The variant 4 of hnRPUL1, which is used in this study, is shorter at the N-terminus without SAP motif compared to variant 1. To determine which domain is responsible for the recruitment of hnRPUL1 (variant 4), we constructed several EGFP-tagged hnRPUL1 deletion mutants and detected the recruitment of these deletion mutants (Figure 2A). As shown in Figure 2B, the C-terminus of hnRPUL1 without RGG motifs is recruited to DNA damage sites, but the N-terminus with SPRY motif is not, whereas the RGG motif shows a weak recruitment and the C-terminus including RGG motif is most strongly recruited. These data suggested that the C-terminus contains domain(s) to be recruited to DSBs and RGG motif supports the recruitment.

### Downregulation of hnRPUL1 Causes Cell Sensitivity to DSBs Damage

**Figure 3:**
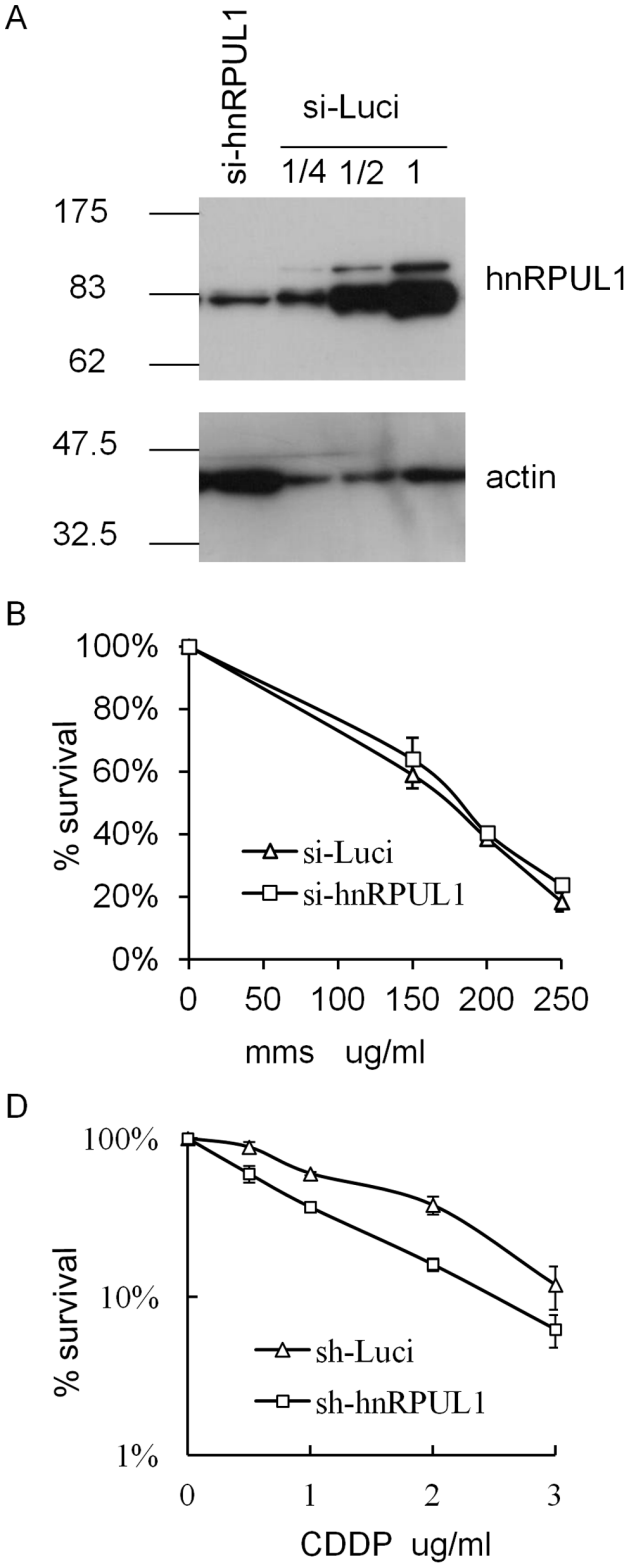
HnRPUL1 knockdown cell showed increased sensitivity to DSB-inducing agents. (A) Immunoblotting analysis of hnRPUL1 stable knockdown cell line and parallel mock knockdown cell line. Actin serves as a loading control for immunoblotting. (B and C) hnRPUL1 knockdown cells cause hypersensitivity to CDDP, but not to MMS. Error bars represent standard errors from two independent experiments.

In light of the above data, we speculated that hnRPUL1 might play an important role in response to DNA damage. Previous works had shown that downregulation of DSBs response proteins increases cell sensitivity to DSBs damage induced by various genotoxic agents. In order to understand the function of hnRPUL1 in responding to DNA damage, we investigated cell sensitivity to DNA damage after downregulation of hnRPUL1. Firstly, we generated a stable hnRPUL1 knockdown HeLa cell line using a vector-based siRNA approach. Characterization of the established cell line indicates that about 80% knockdown is achieved at the protein level (Figure 3A). Downregulation of hnRPUL1 results in slower growth (Figure S2A), but the distribution percentage of cells in each phase is not changed between hnRPUL1 knockdown cell line and a parallel mock knockdown cell (Figure S2B), suggesting that hnRPUL1 is not involved in regulation of cell cycle. Downregulation of hnRPUL1 causes cell sensitivity to X-Ray and CDDP, but not to MMS (Figure S2C, 3B and 3C). HnRPUL1 knockdown increases cell apoptosis after treatment with CDDP (figure S2D). It is consistent with the above data that hnRPUL1 is recruited to DSBs, but not to SSBs sites, and suggests that hnRPUL1 is involved in DSBs damage response and/or repair.

### Recruitment of hnRPUL1 is Dependent on Poly(ADP-ribosyl)ation Mediated by PARP1

**Figure 4:**
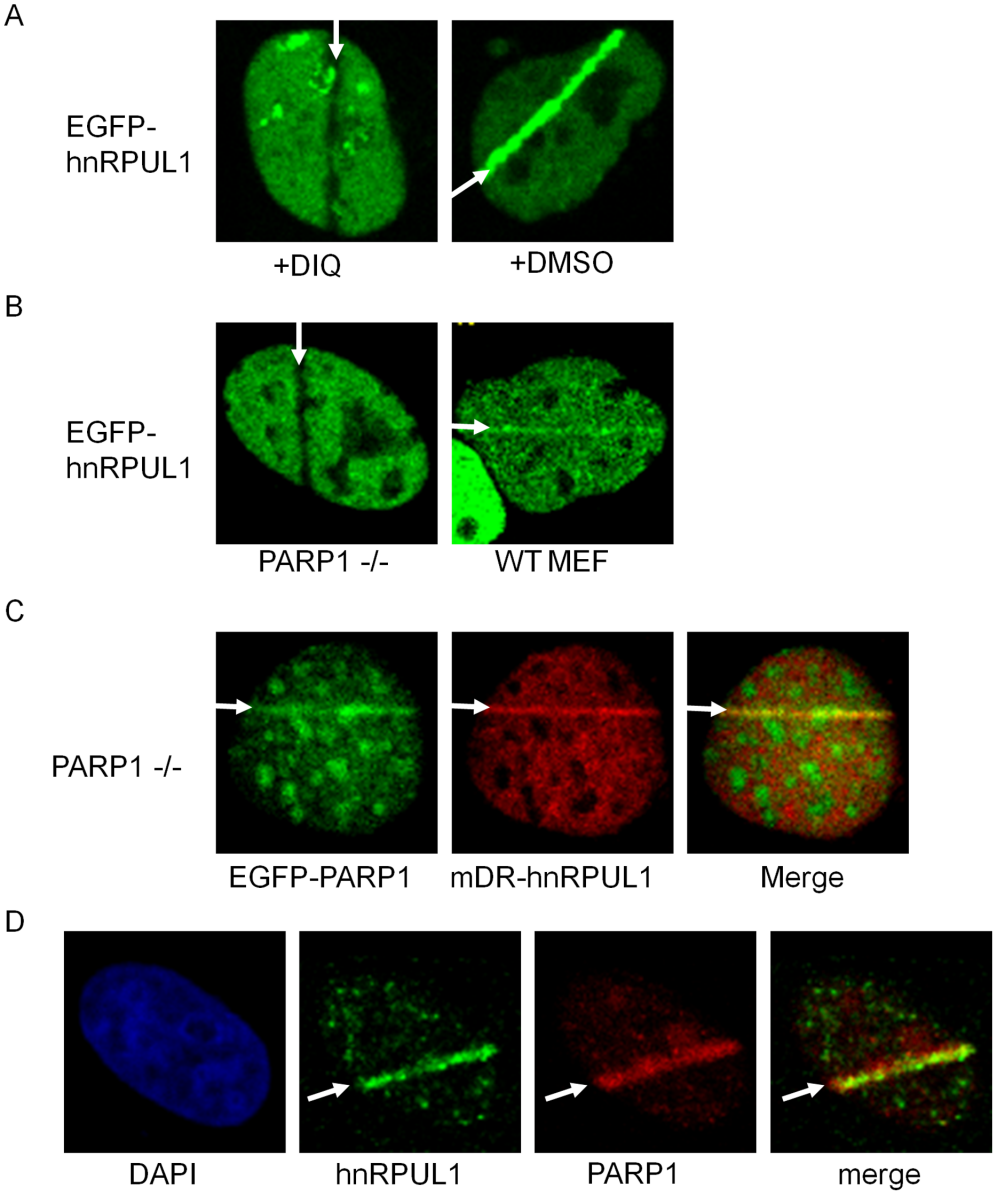
Recruitment of hnRPUL1 is dependent on poly (ADP-ribosyl) ation mediated by PARP1. (A) Recruitment of hnRPUL1 is inhibited by the poly (ADP-ribose) polymerase inhibitor DIQ in HeLa. (B) EGFP-hnRPUL1 is not recruited to irradiated sites in PARP1-deficient MEF cell, but does in PARP1-proficient WT MEF cell. (C) EGFP-hnRPUL1 is recruited to the site of laser-irradiation by coexpression of the EGFP-tagged PARP1 in PARP1-deficient MEF cell. (D) Immunochemical detection of colocalization of endogenous hnRRPUL1 with PARP1 after laser irradiation with BrdU pre-treatment in HeLa cells. Arrows indicate the sites of irradiation.

Previous studies have shown most of DSBs response proteins, such as Ku70/Ku80 and NBS1, to be present at the DSBs sites for a long time (over 1 hr) [18](and our unpublished data). However, hnRPUL1 is rapidly and transiently recruited to DSBs, suggesting that hnRPUL1 plays a function different from classical DSBs response proteins. Poly(ADP-ribosyl)ation is an important post-translational modification of proteins, where the ADP-ribose moiety is transferred to acceptor proteins from NAD^+^ by poly(ADP-ribose)polymerases (PARPs). Poly(ADP-ribosyl)ation is involved in the regulation of many cellular proteins, including recruitment of repair proteins to damage sites [10]. Poly(ADP-ribosyl)ation is a quick and transient response to DNA damage [16], and previous studies have shown that a large number of various hnRNPs can bind to poly(ADP-ribose) (pADPr) [19]. Therefore, we analyzed the effect of poly(ADP-ribosyl)ation on the recruitment of hnRPUL1. As shown in Figure 4A, recruitment of EGFP-hnRPUL1 is inhibited by DIQ (PARP inhibitor) treatment. Because poly(ADP-ribosyl)ation is catalyzed mainly by PARP1, we tested the recruitment of hnRPUL1 in the PARP1−/− MEFs. HnRPUL1 is recruited to damage sites in the wild-type MEF cells, but not in the PARP1−/− MEFs (Figure 4B). Co-expression of mDR-hnRPUL1 (red fluorescent protein-tagged hnRPUL1) and EGFP-PARP1 restored the recruitment of hnRPUL1 in the PARP1−/− MEFs (Figure 4C). Endogenous hnRPUL1 was identified at the irradiated sites and co-localized with PARP1 in HeLa cells (Figure 4D). These data suggest that recruitment of hnRPUL1 to damage sites is dependent on poly(ADP-ribosyl)ation catalyzed by PARP1. Interestingly, EGFP-hnRPUL1 were excluded from the laser-irradiated sites in the PARP1−/− MEFs or in the presence of PARP inhibitor. To analyze whether or not this exclusion was due to fluorescence photobleaching and loss of moblity of EGFP-hnRPUL1 resulted by inhibition of poly(ADP-ribosyl)ation, we performed FRAP analysis. The irradiated region was bleached with low dose laser and the fluorescence recovery of EGFP-hnRPUL1 was detected in presence of DIQ. As shown by Figure S3A, we found a quick fluorescence recovery of EGFP-hnRPUL1. It is in line with previous study [14], suggesting that exclusion of EGFP-hnRPUL1 from DNA damage sites is due to both absence of RNA resulted from local inhibition of transcription at DNA damage site and inhibition of hnRPUL1 recruitment [20]. In line with recruitment of hnRPUL1 through its C-terminus (508–756), recruitment of hnRPUL1 C-terminus (508–756) is also inhibited by PARP1 inhibitor or in the PARP1^−/−^ MEFs cells (Figure S3B and S3C).

### HnRPUL1 is Associated with PARP1 and Affects the Recruitment of PARP1

**Figure 5:**
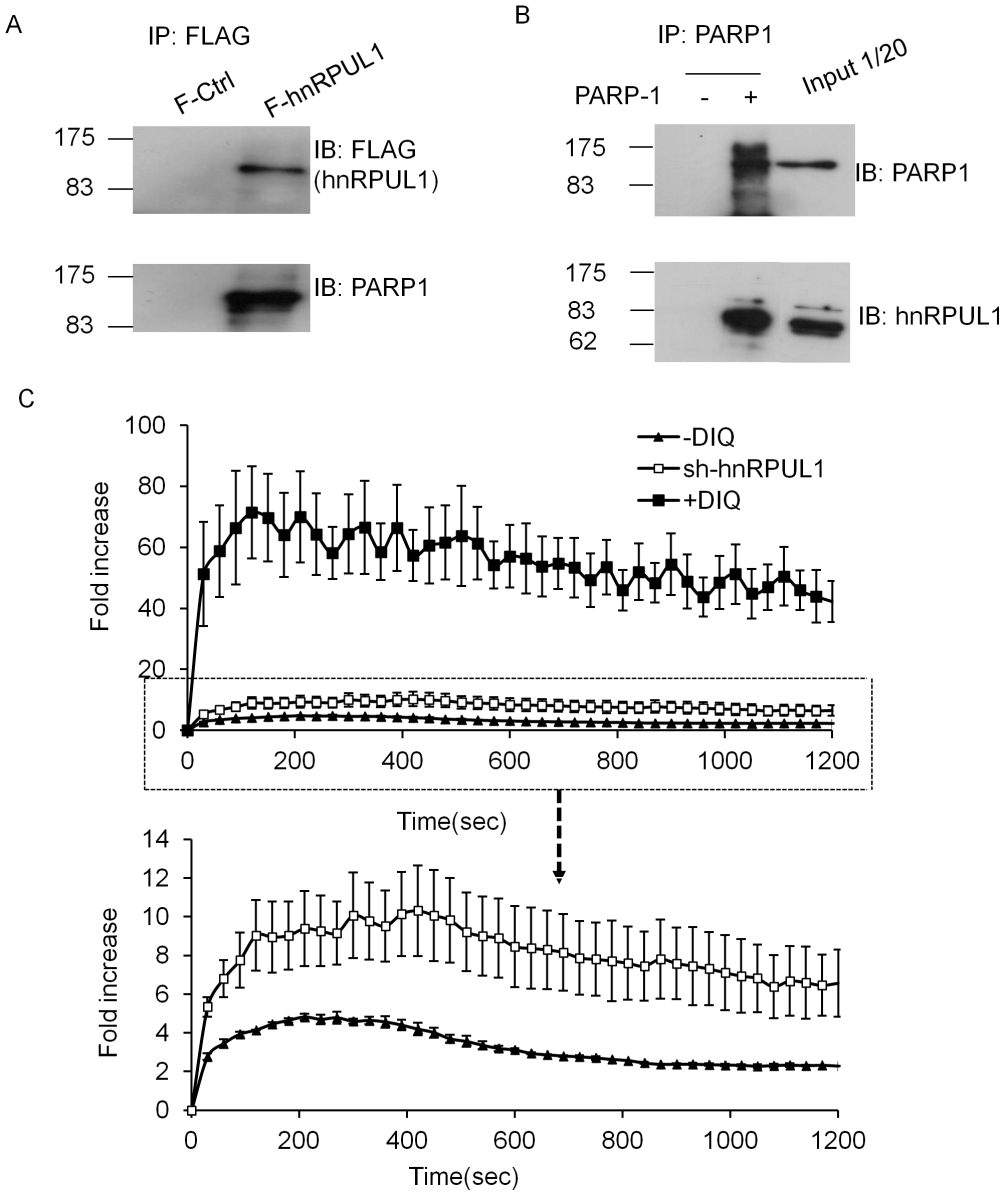
HnRPUL1 is associated physically with PARP1 and influences recruitment of PARP1 to laser-induced DNA damage sites. (A) pcDNA5/FRT vector expressing FLAG-HA tagged hnRPUL1 was transfected into Flp-In-293 cells, and a clone stably expressing FLAG-HA-hnRPUL1 (F-hnRPUL1) was isolated and used for coimmunoprecipitation by anti-FLAG M2 affinity gel. A clone stably expressing FLAG-HA tag alone (F-Control) was used as negative control. (B) PARP1 immunoprecipitates from 293 cell contain hnRPUL1. (C) Quantitative evaluation of EGFP-PARP-1 recruitment kinetics in the absence and presence of the PARP inhibitor DIQ, and in hnRPUL1 knockdown cell. Inhibition of PARP activity promotes prominently recruitment of PARP1 to microirradiated sites, in slimily, recruitment of PARP1 is also increased in hnRPUL1 knockdown cell. Error bars represent standard errors from four independent experiments.

Having shown that the recruitment of hnRPUL1 to damage site is dependent on poly(ADP-ribosyl)ation catalyzed by PARP1, we investigated whether or not hnRPUL1 is associated with PARP1. We generated a 293 cell line stably expressing FLAG-tagged hnRPUL1. HnRPUL1 was immunoprecipitated from 293 cells extracts by using anti-FLAG antibody and the precipitated proteins were analyzed by western blotting with anti-PARP1 antibody. As shown in Figure 5A, PARP1 was indeed co-immunoprecipitated with FLAG-hnRPUL1, but was not present in the control immonoprecipitant. Furthermore, hnRPUL1 could also be coimmunoprecipitated with PARP1 from the 293 cells extracts using anti-PARP1 antibody (Figure 5B). These data indicated that hnRPUL1 is associated with PARP1. Next, we analyzed that which domain of hnRPUL1 is associated with PARP1. As shown in Figure S4A, PARP1 is coimmunoprecipitated with C-terminus (508–756) of hnRPUL1, but not N-terminus (1–507), which is in good agreement with recruitment of hnRPUL1 mediated by its C-terminus (508–756). Furthermore, we found that hnRPUL1 is ribosylated (Figure S4B). It is in accordance with the recruitment of hnRNPUL1 depended on riboyslation. In order to test whether or not hnRPUL1 has an effect on PARP1, we examined the recruitment of PARP1 in hnRPUL1 knockdown cells. We found that recruitment of GFP-tagged PARP1 at laser-induced DNA damage sites is enhanced (Figure S5). Moreover, we analyzed the influence of poly(ADP-ribosyl)ation on the recruitment of PARP1 itself. Interestingly, recruitment of PARP1 is clearly enhanced in the presence of PARP inhibitor DIQ and 3AB (Figure S5). Since the function of PARP1 at DNA damage sites is poly(ADP-ribosyl)ation, we consider that the enhanced recruitment of PARP1 is a consequence of feedback-regulation for effective poly(ADP-ribosyl)ation. Quantitative evaluation of live cell experiments revealed that hnRPUL1 knockdown enhances the recruitment of PARP1 efficiency (Figure 5C), Further more, transient recruitment of hnRPUL1 to DSBs sites is in accord with transient response of Poly(ADP-ribosyl)ation to DNA damage. For these reasons, hnRPUL1 plays a possible role as a coeffector of PARP1 in PAR synthesis.

### HnRPUL1 Affects PARP1 Expression by Transcriptional Regulation

**Figure 6:**
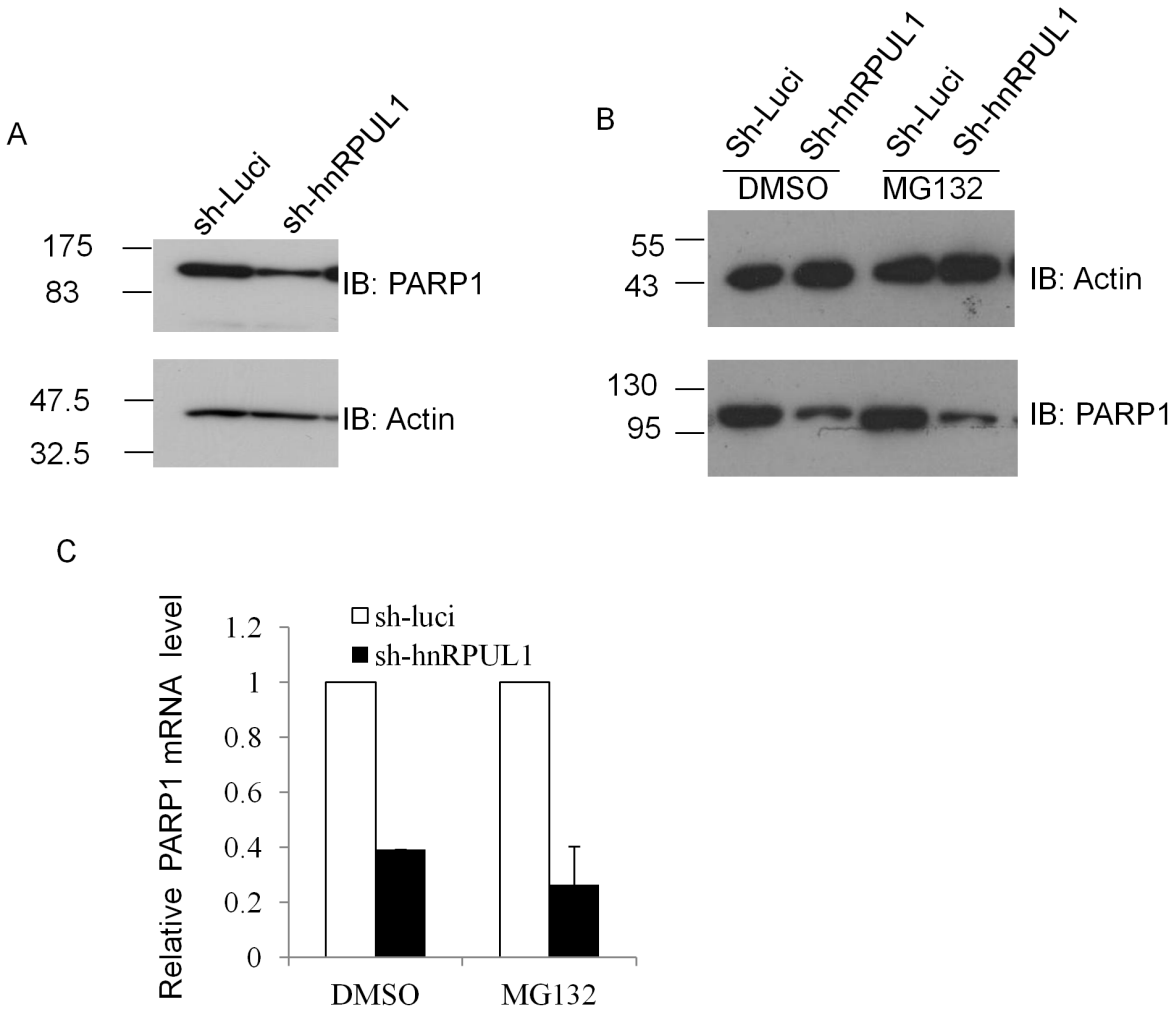
HnRPUL1 knockdown reduces PARP1 expression by transcriptional regulation. (A) The level of PARP1 proteins is analyzed by western blotting of total extracts from hnRPUL1 knockdown cell and parallel mock knockdown cell. (B)Analysis of the level of PARP1 proteins after MG132 treatment. (C) The level of PARP1 mRNA is analyzed by quantitative real-time PCR in hnRPUL1 knockdown cell line and a parallel mock knockdown cell with and without MG132 treatment. Error bars represent standard errors from three independent experiments.

In order to further test the effect of hnRPUL1 on PARP1, we investigated whether or not downregulation of hnRPUL1 has effect on expression of PARP1. As shown in Figure 6A, the level of PARP1 protein was reduced after hnRPUL1 knockdown. This data indicates that hnRPUL1 plays a role in regulating PARP1 expression. It is well-known that level of protein expression is regulated by two pathways: transcription regulation and proteolytic degradation by the proteasome. For further understanding the mechanism of hnRPUL1 regulating PARP1 expression, we first checked whether or not proteolytic degradation by the proteasome is involved in hnRPUL1 regulating PARP1 expression. As shown in Figure 6B, the level of PARP1 protein is still reduced after knockdown of hnRPUL1 when proteolytic degradation is inhibited by MG132, and this suggested that downregulation of PARP1 after knockdown of hnRPUL1 is involved in transcription regulation. Then we checked the mRNA level of PARP1 by quantitative real-time polymerase chain reaction, as shown in figure 6C, and we found mRNA level of PARP1 is significantly reduced after knockdown of hnRPUL1. In order to avoid that downregulation of PARP1 is due to an “off-target” effect of the shRNA, we generated another stable hnRPUL1 knockdown HeLa cell line targeting difference site. Both protein level and mRNA level of PARP1 were also reduced in this cell (Figure S6). Furthermore, previous study has shown that hnRPUL1 has a role in RNA regulation like other hnRNPs [3]. Therefore, these data suggested that hnRPUL1 plays a key role in the transcriptional regulation of PARP1.

## Discussion

In the present work, we have revealed a function of hnRPUL1 proteins in DSBs response. Our data demonstrated that hnRPUL1 is associated with PARP1 and recruited to DSBs sites in a PARP1-mediated poly(ADP-ribosyl)ation-dependent manner. In turn, hnRPUL1 knockdown enhances the recruitment of PARP1 to DSBs sites. Specifically, we showed that hnRPUL1 is also implicated in the transcriptional regulation of PARP1 gene. Thus, we have shown a newly identified role for hnRPUL1 in DNA damage response and repair that correlates with PARP1.

HnRPUL1 was first identified as the interacting protein with adenovirus early protein E1B-55 kDa, and sequence analysis suggested it belongs to hnRNP family [3]. HnRNPs are multifunctional proteins, which were described as nuclear RNA-binding proteins. In addition to binding RNA polymerase II transcripts and regulating RNA metabolism, hnRNPs also bind DNA and play a role in telomere elongation, chromatin remodeling and transcription [1], [2]. Furthermore, recent studies have shown that hnRNPs are also involved in DNA damage response and repair. HnRNP B1 interacts with DNA-PK complex and impairs DSBs repair through inhibiting DNA-PK activity [21]. HnRNP C1/C2 binds to chromatin in a DNA-damage-dependent manner [19], and it also binds to Ku and is phosphorylated by DNA-PK in an RNA-dependent manner, thus suggesting hnRNP C1/C2 may participate in DNA DSBs damage response and repair [22]. HnRNP K, as a cofactor of p53, regulates the transcription in response to DNA damage. Moreover, hnRNP G is able to bind to DNA ends and promotes the fidelity of DNA end-joining through the pathway medicated by p53 [23]. Our work provided a new evidence indicating that hnRNPs are implicated in DNA damage response and repair.

Recent study has shown that hnRPUL1 is recruited to DNA damage sites induced by laser [14]. It should be noted that laser can induce base damage, SSBs and DSBs. Here, we demonstrated that hnRPUL1 is recruited to DSBs sites, but not to SSBs sites. Although hnRPUL1 is associated with DSB sensor complex MRE11-RAD50-NBS1 (MRN) and recruited to DNA damage sites dependent on MRN [14], we found that hnRPUL1 is also associated with PARP1 and recruited to DSBs sites in a PARP1-mediated poly(ADP-ribosyl)ation-dependent manner. It suggests that the function of hnRPUL1 in DSBs response is regulated by both PARP1 and MRN. Previous studies have shown PARP1 is involved in DSB repair through both NHEJ and HR by different signaling pathways [24], [25], [26]. PARP1 is the first protein recruited to DNA damage sites and modulates ATM signaling pathway by poly(ADP-ribosyl)ation [27]. It should be noted that PARP1 is associated with MRE11, and is required for recruitment of MRN [28]. Therefore, our data on the PARP1-mediated poly(ADP-ribosyl)ation-dependent recruitment of hnRPUL1 suggests a model that MRN proteins are recruited to DSBs following the recruitment of hnRPUL1 by activation of PARP1 at DSBs. Interestingly, hnRNPG (also known as RBMX), another hnRNP protein, was shown to be associated with PARP1 and recruited to DSBs sites in a PARP1-dependent manner [29] (and our unpublished data). Both hnRPUL1 and hnRNPG have been shown to promote HR by different pathways [14], [29]. Take together, hnRNPs play an important role in DNA damage response by activating different signaling pathway, and are generally regulated by PARP1.

Another function of hnRPUL1 is involved in RNA metabolism, which is still incompletely understood. In this study, our data demonstrating that hnRPUL1 knockdown reduces the mRNA level of PARP1 provides the possibility that hnRPUL1 affects DNA damage response indirectly by transcriptional regulation. In conclusion, the results presented in this study identified a new function of hnRPUL1 which plays a role in the maintenance of genomic integrity through interaction with PARP1 and transcriptional regulation of PARP1 expression.

## Supporting Information

Figure S1 **Recruitment of hnRPUL1 to DSBs sites.** (A) Recruitment kinetics of EGFP-tagged hnRPUL1 after 500 scans with 405 nm laser with BrdU pre-treatment in HeLa cells. Arrows indicate the sites of irradiation. (B) Immunochemical detection colocalization of endogenous hnRRPUL1 with γH2AX after laser irradiation with BrdU pre-treatment in HeLa cells. Arrows indicate the sites of irradiation.
(TIF)

Figure S2 **HnRPUL1 knockdown results in cell growth inhibition and increases cell apoptosis after CDDP treatment.** (A) The growth curve of control and knockdown HeLa cells. Viable cells were counted at different times after initial seeding of 1×10^5^ cells. (B) Cell cycle analysis of hnRPUL1 knockdown cell line and a parallel mock knockdown cell by flow cytometry. (C) HnRPUL1 knockdown cell cause hypersensitivity to X-Ray. (D) Apoptosis analysis by flow cytometry after CDDP treatment.
(TIF)

Figure S3 **Recruitment of hnRPUL1 C-terminus (508–756) is dependent on poly (ADP-ribosyl) ation.** (A) FRAP analysis of EGFP-hnRPUL1 after DIQ treatment. A region of interest was selected and photobleached for 20 frames with 405 nm laser set to maximum power at 100% transmission. Before and after bleaching, confocal image series were recorded. The line shows the bleached microirradiated site. (B) And (C) recruitment of hnRPUL1 C-terminus (508–756) is inhibited by PARP1 inhibitor DIA or in the PARP1^−/−^ MEFs cells.
(TIF)

Figure S4 **HnRPUL1 is associated with PARP1 via C-terminus (508–756) and is ribosylated in cells.** (A) N-terminus (1–507) and C-terminus (508–756) of hnRPUL1 with Flag tag were traniently expressed in HeLa cells, whole cell lysates were Immunoprecipitated by anti-FLAG antibody. Blank vector as a control. (B) FLAG-HA-hnRPUL1 (F-hnRPUL1) stably expressed in 293 cells was coimmunoprecipitated by anti-FLAG antibody. Immunoprecipitated hnRPUL1 was blotted by anti-FLAG antibody and anti-pAR antibody.
(TIF)

Figure S5 **EGFP-PARP-1 recruitment kinetics in the absence or presence of the PARP inhibitor DIQ, 3AB and in hnRPUL1 knockdown cell.**
(TIF)

Figure S6 **HnRPUL1 knockdown reduces PARP1 expression both in protein and mRNA level.** (A) Immunoblotting analysis of hnRPUL1 stable knockdown cell line and parallel mock knockdown cell line. Actin serves as a loading control for immunoblotting. Stable hnRPUL1 knockdown HeLa cell line was generated by targeting nucleotide sequence (5-GCAACTATATCCTAGATCAGA). (B) The level of PARP1 mRNA is analyzed by quantitative real-time PCR in hnRPUL1 knockdown cell line and a parallel mock knockdown cell. Error bars represent standard errors from three independent experiments.
(TIF)

Text S1 **Supplementary Methods and figure legend.**
(DOC)
