# Supplementary figures and images for: The Role of hnRPUL1 Involved in DNA Damage Response Is Related to PARP1
Source: PLoS One. 2013 Apr 5;8(4):e60208. doi: 10.1371/journal.pone.0060208 (PMC3618279; doi:10.1371/journal.pone.0060208)

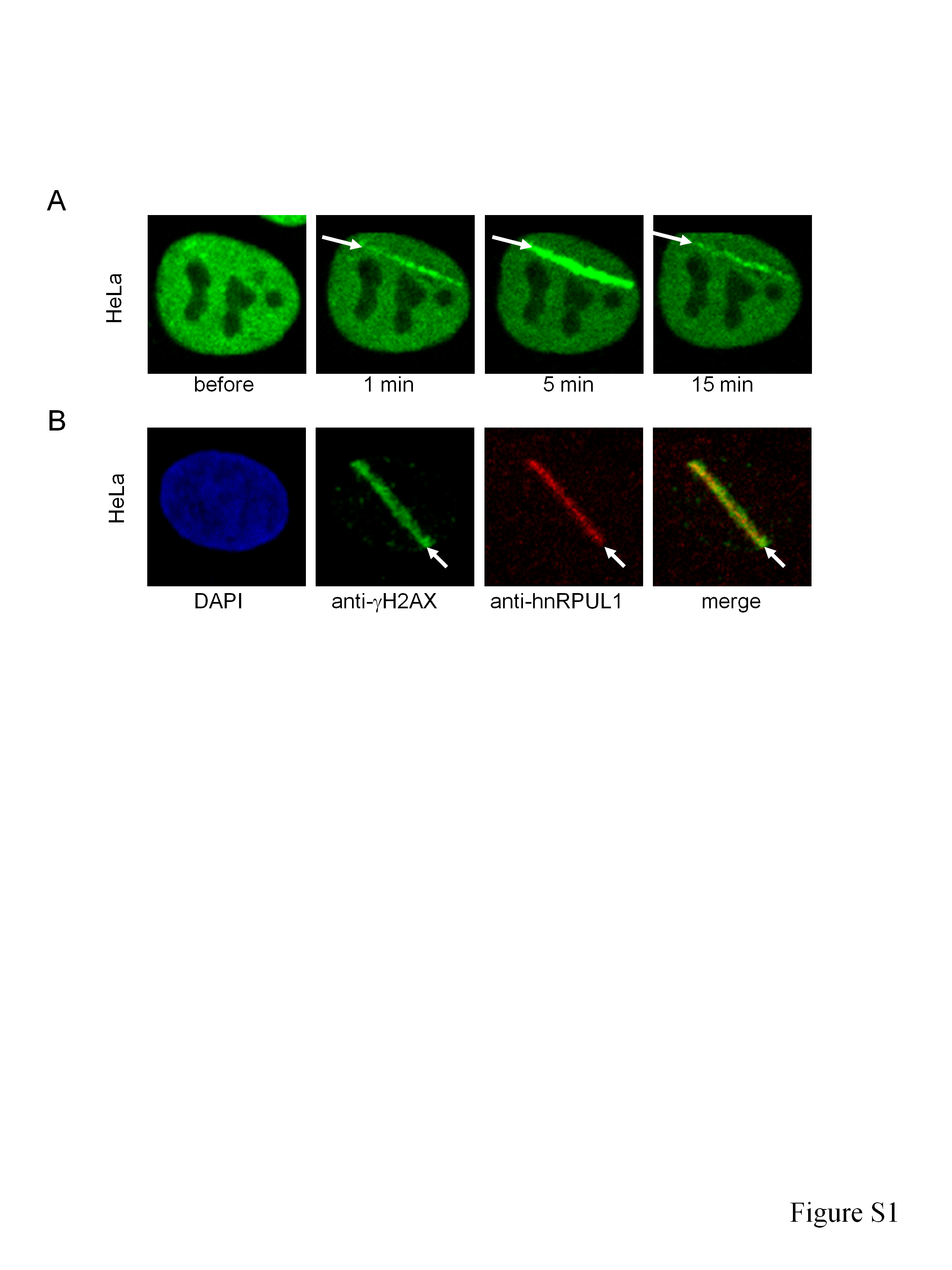

Supplement: Figure S1 — Recruitment of hnRPUL1 to DSBs sites. (A) Recruitment kinetics of EGFP-tagged hnRPUL1 after 500 scans with 405 nm laser with BrdU pre-treatment in HeLa cells. Arrows indicate the sites of irradiation. (B) Immunochemical detection colocalization of endogenous hnRRPUL1 with γH2AX after laser irradiation with BrdU pre-treatment in HeLa cells. Arrows indicate the sites of irradiation. (TIF) [file pone.0060208.s001.tif]

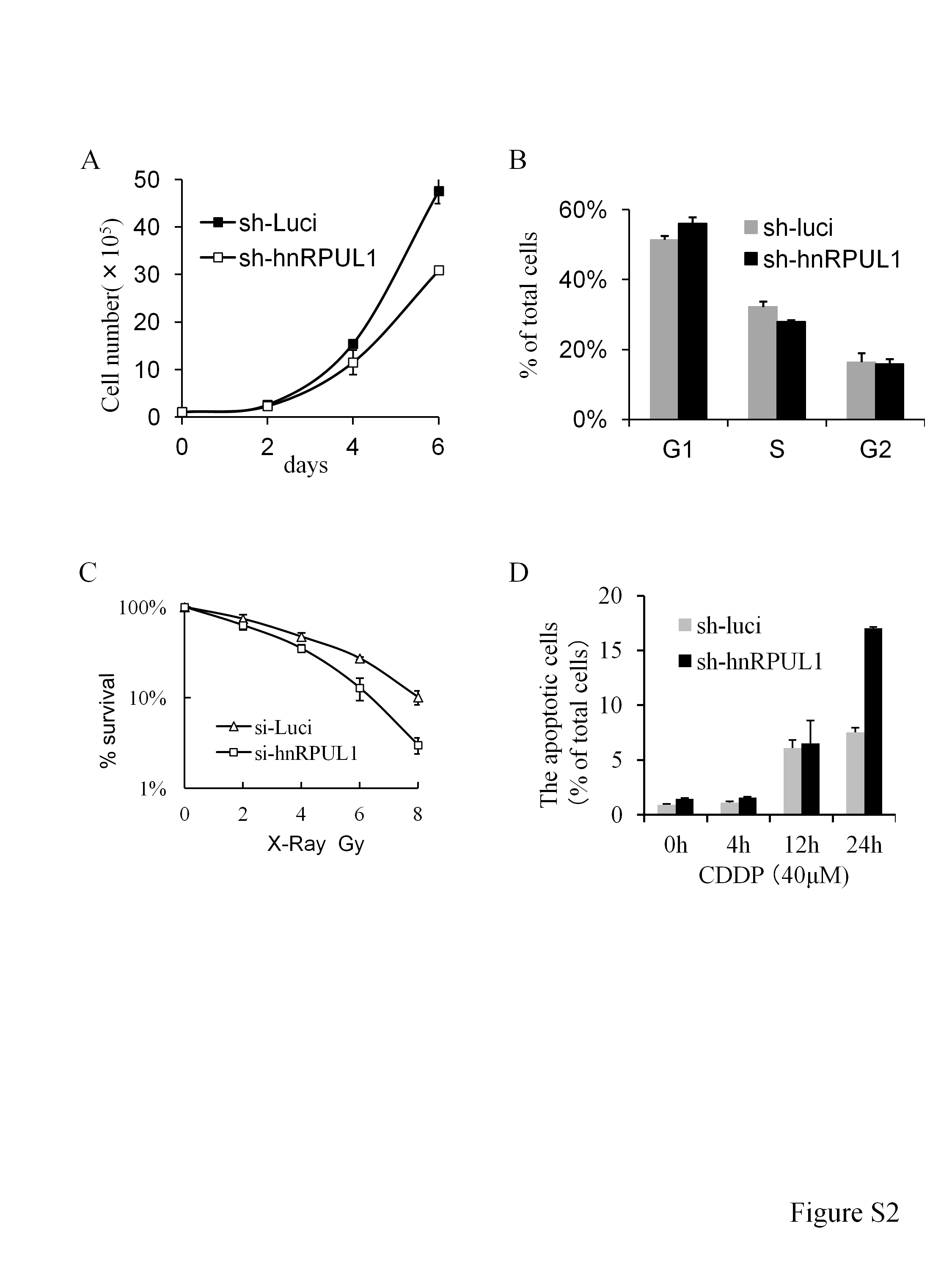

Supplement: Figure S2 — HnRPUL1 knockdown results in cell growth inhibition and increases cell apoptosis after CDDP treatment. (A) The growth curve of control and knockdown HeLa cells. Viable cells were counted at different times after initial seeding of 1×10^5^ cells. (B) Cell cycle analysis of hnRPUL1 knockdown cell line and a parallel mock knockdown cell by flow cytometry. (C) HnRPUL1 knockdown cell cause hypersensitivity to X-Ray. (D) Apoptosis analysis by flow cytometry after CDDP treatment. (TIF) [file pone.0060208.s002.tif]

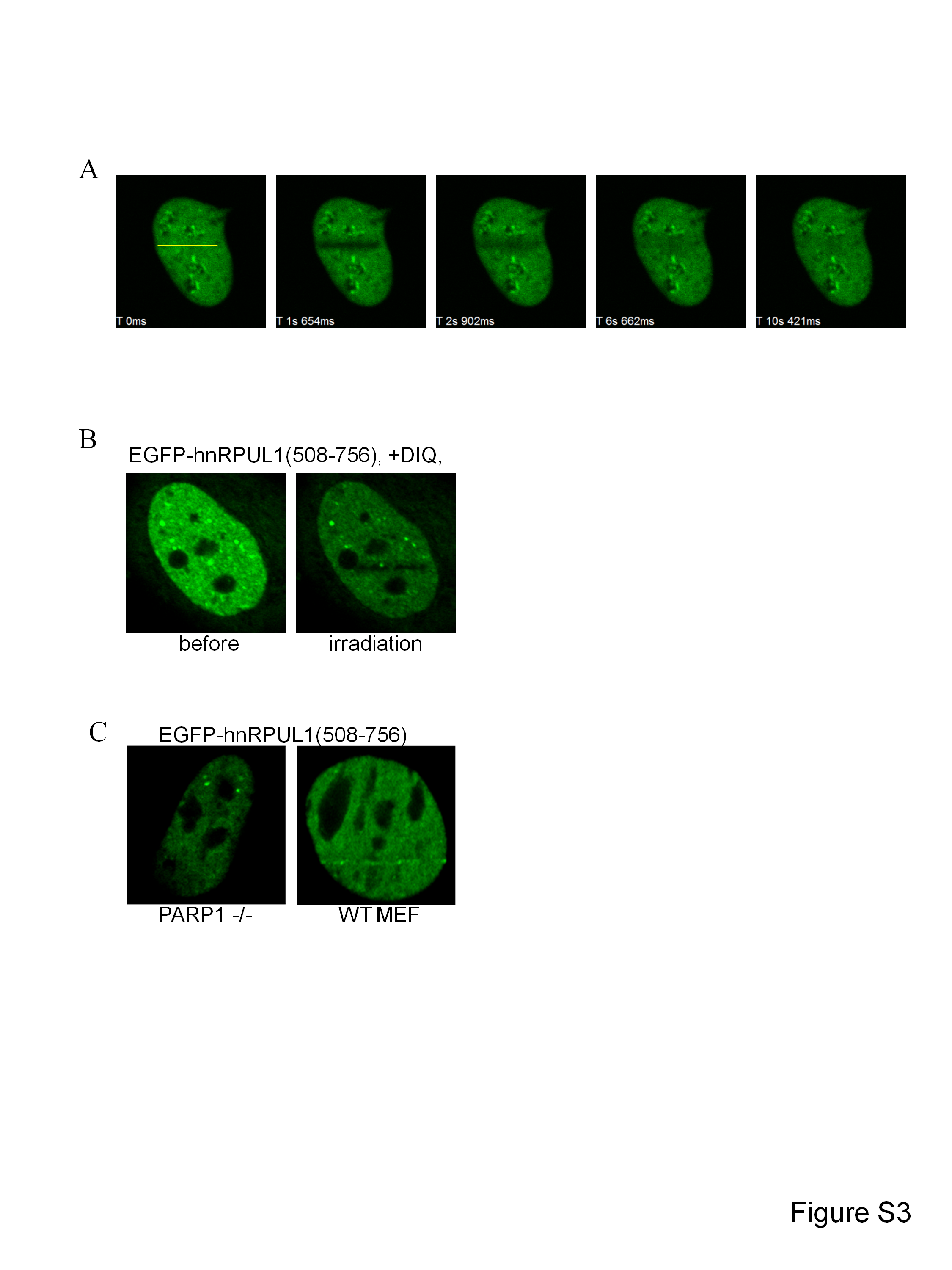

Supplement: Figure S3 — Recruitment of hnRPUL1 C-terminus (508–756) is dependent on poly (ADP-ribosyl) ation. (A) FRAP analysis of EGFP-hnRPUL1 after DIQ treatment. A region of interest was selected and photobleached for 20 frames with 405 nm laser set to maximum power at 100% transmission. Before and after bleaching, confocal image series were recorded. The line shows the bleached microirradiated site. (B) And (C) recruitment of hnRPUL1 C-terminus (508–756) is inhibited by PARP1 inhibitor DIA or in the PARP1−/− MEFs cells. (TIF) [file pone.0060208.s003.tif]

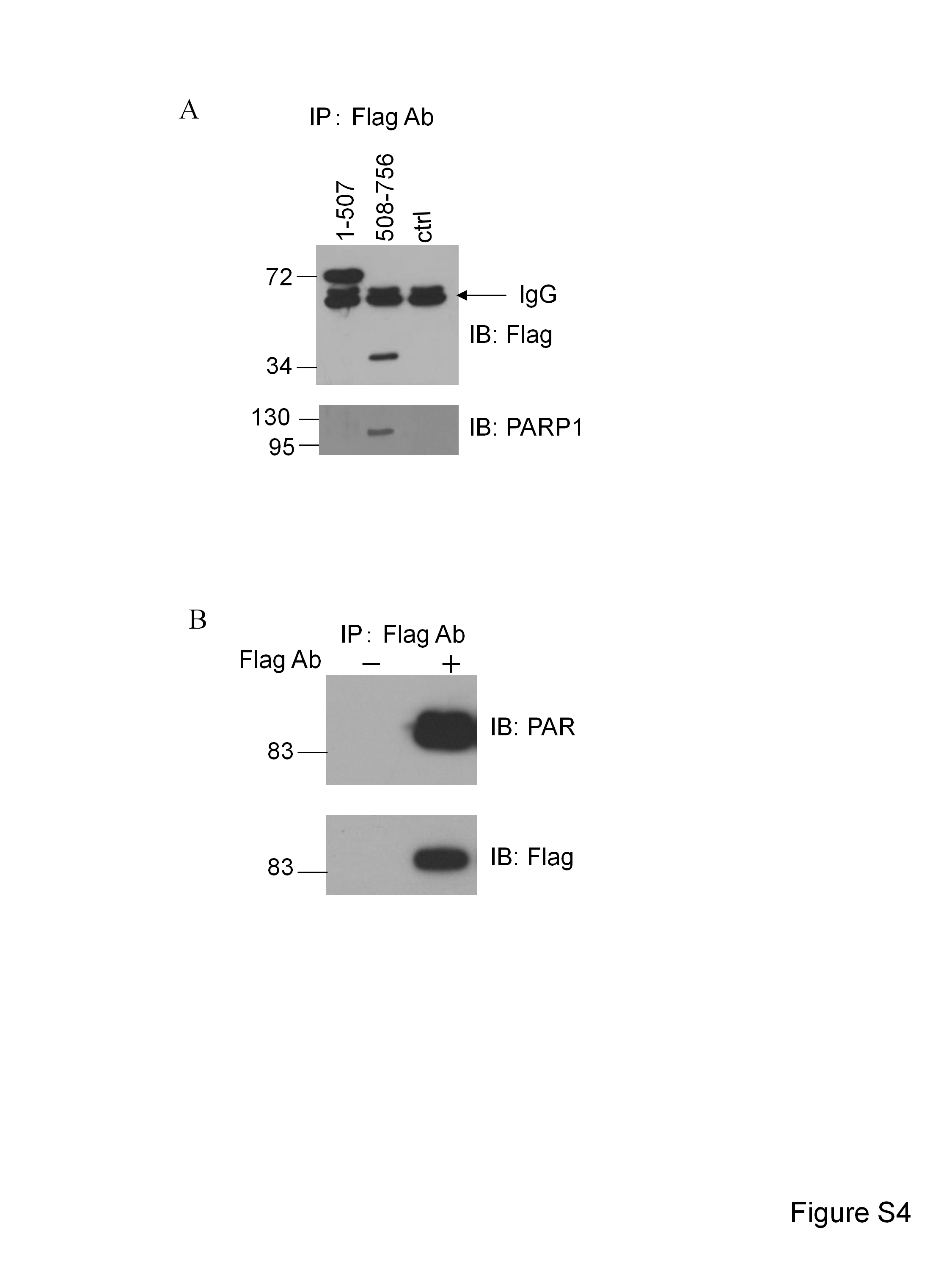

Supplement: Figure S4 — HnRPUL1 is associated with PARP1 via C-terminus (508–756) and is ribosylated in cells. (A) N-terminus (1–507) and C-terminus (508–756) of hnRPUL1 with Flag tag were traniently expressed in HeLa cells, whole cell lysates were Immunoprecipitated by anti-FLAG antibody. Blank vector as a control. (B) FLAG-HA-hnRPUL1 (F-hnRPUL1) stably expressed in 293 cells was coimmunoprecipitated by anti-FLAG antibody. Immunoprecipitated hnRPUL1 was blotted by anti-FLAG antibody and anti-pAR antibody. (TIF) [file pone.0060208.s004.tif]

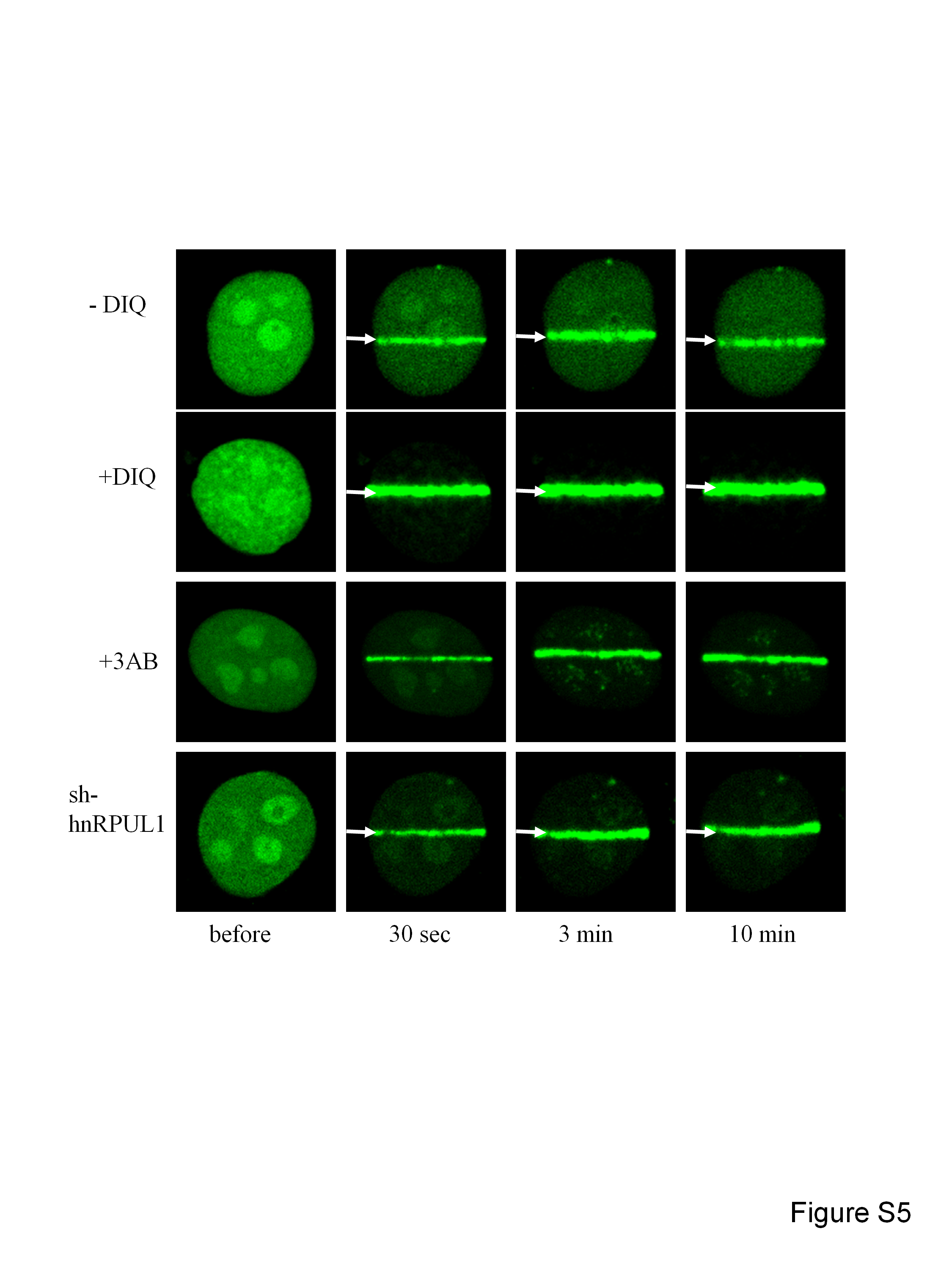

Supplement: Figure S5 — EGFP-PARP-1 recruitment kinetics in the absence or presence of the PARP inhibitor DIQ, 3AB and in hnRPUL1 knockdown cell. (TIF) [file pone.0060208.s005.tif]

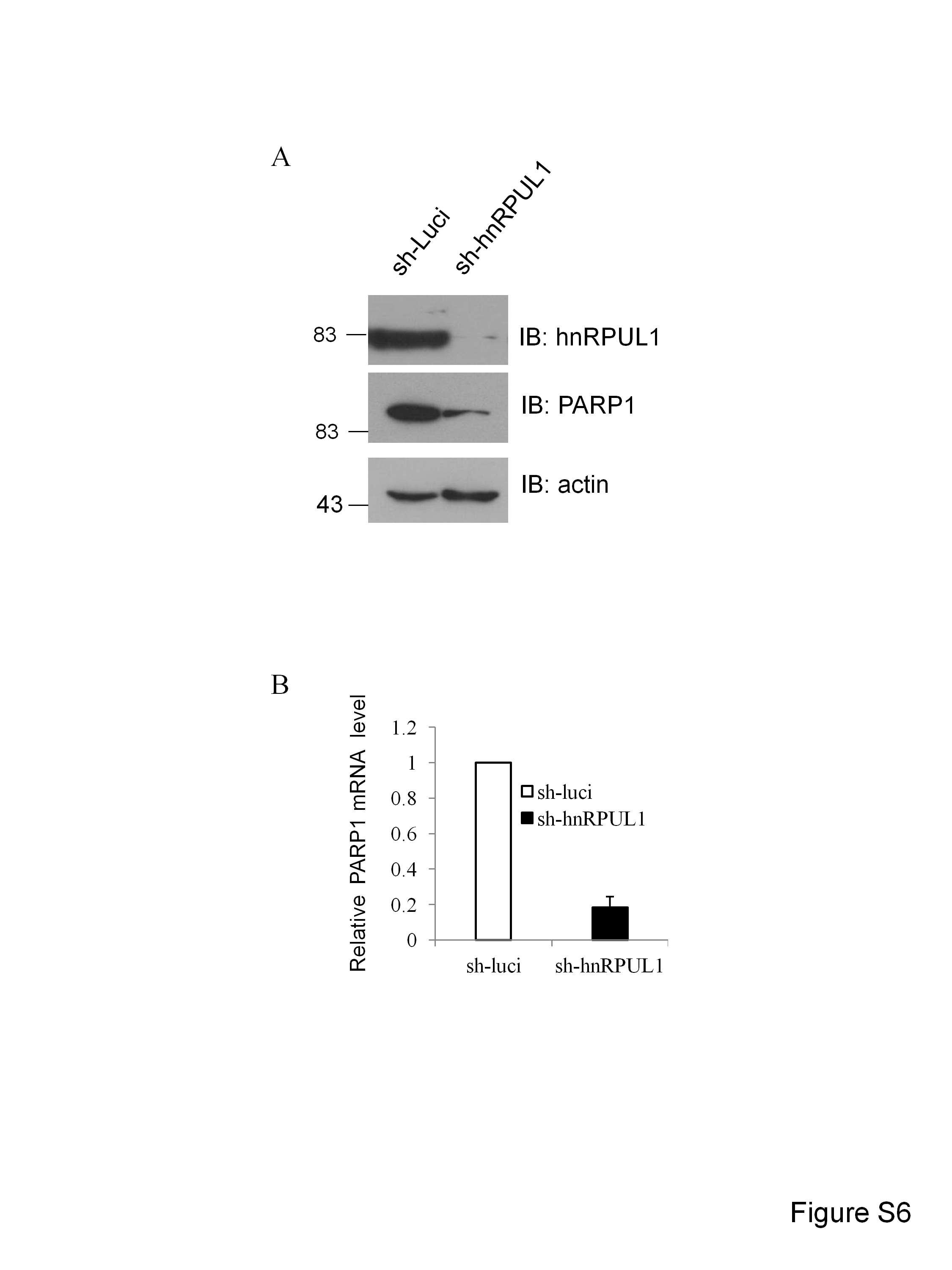

Supplement: Figure S6 — HnRPUL1 knockdown reduces PARP1 expression both in protein and mRNA level. (A) Immunoblotting analysis of hnRPUL1 stable knockdown cell line and parallel mock knockdown cell line. Actin serves as a loading control for immunoblotting. Stable hnRPUL1 knockdown HeLa cell line was generated by targeting nucleotide sequence (5-GCAACTATATCCTAGATCAGA). (B) The level of PARP1 mRNA is analyzed by quantitative real-time PCR in hnRPUL1 knockdown cell line and a parallel mock knockdown cell. Error bars represent standard errors from three independent experiments. (TIF) [file pone.0060208.s006.tif]
